# Creating single-atom Pt-ceria catalysts by surface step decoration

**DOI:** 10.1038/ncomms10801

**Published:** 2016-02-24

**Authors:** Filip Dvořák, Matteo Farnesi Camellone, Andrii Tovt, Nguyen-Dung Tran, Fabio R. Negreiros, Mykhailo Vorokhta, Tomáš Skála, Iva Matolínová, Josef Mysliveček, Vladimír Matolín, Stefano Fabris

**Affiliations:** 1 Charles University in Prague, Faculty of Mathematics and Physics, V Holešovičkách 2, Prague 18000, Czech Republic; 2 CNR-IOM DEMOCRITOS, Istituto Officina dei Materiali, Consiglio Nazionale delle Ricerche, Via Bonomea 265, Trieste 34136, Italy; 3 SISSA, Scuola Internazionale Superiore di Studi Avanzati, Via Bonomea 265, Trieste 34136, Italy

## Abstract

Single-atom catalysts maximize the utilization of supported precious metals by exposing every single metal atom to reactants. To avoid sintering and deactivation at realistic reaction conditions, single metal atoms are stabilized by specific adsorption sites on catalyst substrates. Here we show by combining photoelectron spectroscopy, scanning tunnelling microscopy and density functional theory calculations that Pt single atoms on ceria are stabilized by the most ubiquitous defects on solid surfaces—monoatomic step edges. Pt segregation at steps leads to stable dispersions of single Pt^2+^ ions in planar PtO_4_ moieties incorporating excess O atoms and contributing to oxygen storage capacity of ceria. We experimentally control the step density on our samples, to maximize the coverage of monodispersed Pt^2+^ and demonstrate that step engineering and step decoration represent effective strategies for understanding and design of new single-atom catalysts.

## Abstract (web summary)

Single metal atoms promise high catalytic performances, but their implementation in future systems depends on an understanding of how their underlying support medium can offer stabilization. Here, the authors investigate Pt^2+^ on ceria to elucidate this important fundamental consideration.

## Graphical abstract

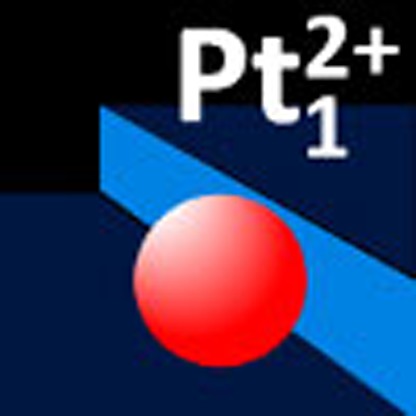

Single-atom catalysts represent the limiting realization of supported metal catalysts with metal load ultimately dispersed as single atoms^1^,^2^. This maximizes the utilization of supported metals and helps development of sustainable catalytic technologies for renewable energies and environmental applications with reduced precious metal contents^3^,^4^. A central prerequisite for understanding and knowledge-based design of single-atom catalysts is the identification of specific adsorption sites on catalyst supports that provide the stabilization of single metal atoms under reaction conditions at elevated temperatures and pressures. For oxide supports, understanding specific adsorption sites presently concentrates on low-index oxide facets^5^,^6^,^7^,^8^,^9^. Single-atom catalysts are, however, nanostructured large-area materials; thus, a question arises whether single supported atoms can be stabilized at defect sites of nanostructured oxide supports.

Highly dispersed platinum (Pt) ions on ceria qualify as single-atom catalysts^2^ and hold a promise of radical reduction of Pt load in critical large-scale catalytic applications—hydrogen production^3^, three-way catalytic converters^10^ and fuel cells^11^. Ceria surfaces provide a limited amount of low coordinated surface sites where Pt^2+^ ions can adsorb and remain stable in real applications^2^,^3^,^10^,^11^. Recent studies on large-area ceria samples identify the necessity of nanostructuring the ceria substrates for obtaining supported Pt^2+^ ions^2^,^4^ and propose a square-planar PtO_4_ unit as a Pt^2+^-containing surface moiety^4^. In the present model study on the single crystalline CeO_2_(111) surface, we demonstrate that single-ion dispersions of Pt^2+^ are stabilized at monolayer (ML)-high ceria step edges. Pt^2+^ ions at step edges are located in PtO_4_ units that can be considered the elementary building blocks of Pt^2+^/ceria single-atom catalysts. The PtO_4_ units incorporate excess O and can act as oxygen source for redox reactions. Besides clarifying the nature of Pt^2+^ stabilization on ceria, our study demonstrates the importance of step edges—the most common surface defects on oxide supports^12^—for single-atom catalyst stabilization. We experimentally adjust the step density on the ceria supports for maximizing the load of monodispersed Pt^2+^ ions. This identifies step engineering^13^ and step decoration^14^,^15^ as advanced techniques for designing new single-atom catalysts.

## Results

### Pt deposits on highly defined CeO_2_(111) surfaces

**Figure 1:**
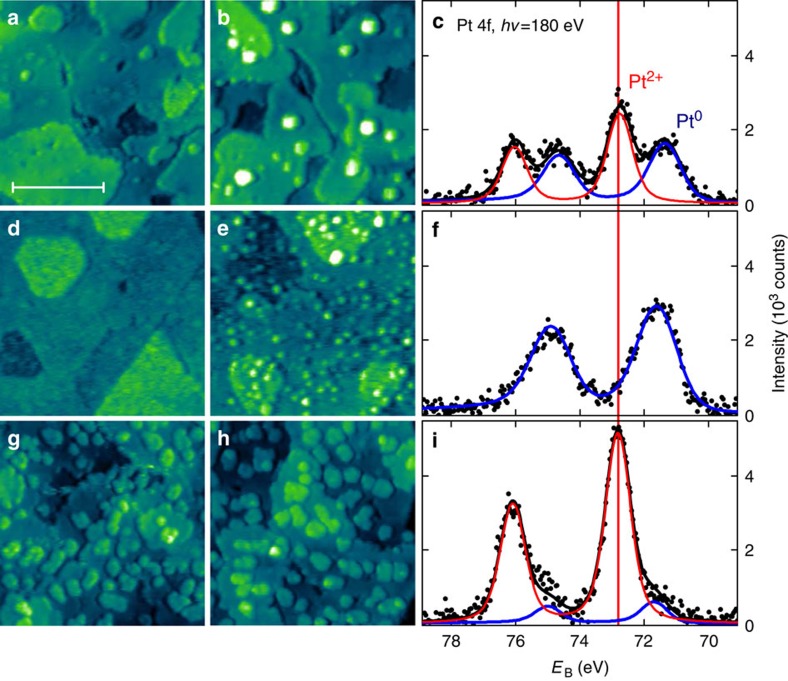
Nucleation of Pt and stabilization of Pt^2+^ on ceria surfaces containing controlled amount of surface defects. (**a**–**c**) CeO_2_(111) surface with low density of surface oxygen vacancies and ML-high steps. (**d**–**f**) CeO_1.7_ surface with increased density of surface oxygen vacancies. (**g**–**i**) CeO_2_(111) surface with increased density of ML-high steps. (**a**,**d**,**g**) STM images of clean surfaces before deposition of Pt. (**b**,**e**,**h**) STM images after deposition of 0.06 ML Pt and annealing at 700 K in UHV. All STM images 45 × 45 nm^2^, tunnelling current 25–75 pA, sample bias voltage 2.5–3.5 V. Scale bar, 20 nm (**a**). (**c**,**f**,**i**) PES spectra of the Pt deposit after annealing. All PES spectra were acquired with photon energy *hν*=180 eV (black points). Fits indicate metallic (Pt^0^, blue line) and ionic (Pt^2+^, red line) contributions to Pt 4f signal. *E*_B_ is the photoelectron binding energy.

The experiments were performed on model CeO_2_(111) surfaces prepared as 20 to 40 Å thick ceria films on Cu(111) using procedures that allow adjusting the density of ML-high steps^16^ and the density of surface oxygen vacancies on ceria surface^17^. On these highly defined surfaces, we deposit 0.06 ML of Pt, anneal at 700 K in ultra-high vacuum (UHV) and observe stabilization of Pt^2+^ species and/or nucleation of Pt clusters with scanning tunnelling microscopy (STM) and with photoelectron spectroscopy (PES). Deposition and annealing of Pt on CeO_2_(111) surfaces containing low concentrations of defects—ML-high steps and surface oxygen vacancies (Fig. 1a)—yield metallic Pt^0^ clusters (Fig. 1b,c) coexisting with ionic Pt^2+^ species (Fig. 1c). To determine whether the charge of the supported Pt species is selectively induced by a specific defect type, we repeat the experiment varying independently the amount of surface O vacancies—up to 0.16 ML, creating CeO_1.7_ surface (Fig. 1d–f)—and the amount of ML-high steps on the CeO_2_(111) surface—up to 0.15 ML (Fig. 1g–i). We observe that surface oxygen vacancies do not promote the dispersion of Pt^2+^ species but lead to small metallic Pt^0^ clusters (Fig. 1e,f)^18^. On the other hand, the increased step density leads to almost complete oxidation of the Pt deposit to Pt^2+^ (Fig. 1h,i) proving that step edges selectively promote the stabilization of Pt^2+^ species. Detailed STM images allow to exclude formation of three-dimensional and two-dimensional PtO_*x*_ clusters (Supplementary Fig. 1), and allow to conclude that Pt^2+^ species are incorporated in the ceria step edges. Nucleation of Pt^0^ clusters and stabilization of Pt^2+^ species represent concurrent processes. Differently to Pt^0^ clusters (Fig. 1b,e), Pt^2+^ species at the step edges are not discernible in empty states STM imaging (*cf*. Fig. 1g,h without and with Pt deposit) because of their electronic structure. STM imaging in occupied states on metal-supported ceria is unavailable^19^.

The possibility to adjust the density of ML-high steps on the model CeO_2_(111) surfaces^16^ allows us to obtain a quantitative correlation between the step density and the amount of Pt^2+^ species. We prepare CeO_2_(111) samples with step density between 0.06 and 0.20 ML^16^ and deposit 0.06 or 0.18 ML Pt at 300 K. Parameters of the prepared samples are summarized in Supplementary Table 1. After annealing at 700 K the amount of Pt stabilized in the form of Pt^2+^ is determined by PES. For quantification, all relevant parameters—the density of ceria steps, deposited amount of Pt and amount of stabilized Pt^2+^—are expressed in ML where 1 ML corresponds to the density of Ce atoms on the CeO_2_(111) surface, that is, 7.9 × 10^14^ cm^−2^. The density of steps is defined as the density of Ce atoms located at the ceria step edges^20^.

**Figure 2:**
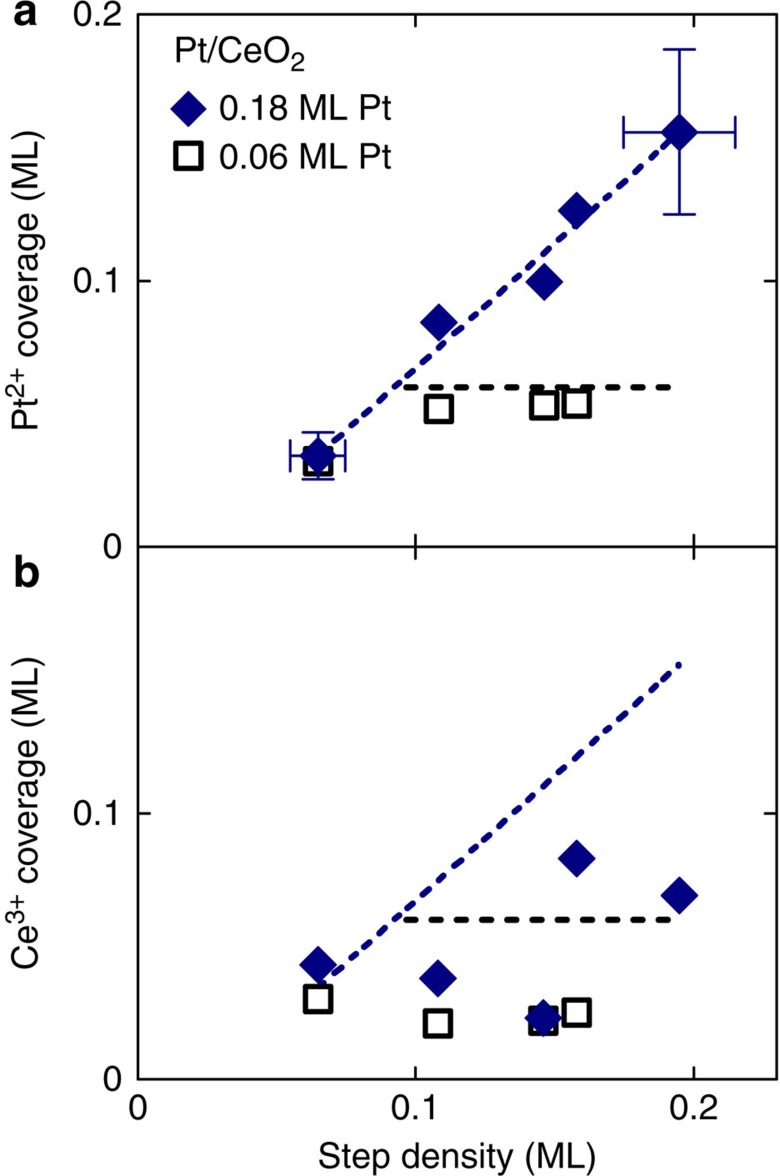
Capacity of stepped CeO_2_(111) surface to accommodate Pt^2+^. (**a**) Amount of Pt^2+^ stabilized on CeO_2_(111) substrates with different density of steps for 0.18 ML (blue symbols) and 0.06 ML (black symbols) of deposited platinum. Pt not stabilized in the form of Pt^2+^ remains metallic. Lines represent guides to the eyes. Blue line is a linear fit of 0.18 ML Pt data. Black line represents the maximum achievable amount of Pt^2+^ in the case of 100% oxidation of Pt for 0.06 ML of deposited Pt. (**b**) Reduction of the ceria surface accompanying the stabilization of Pt^2+^ ions determined by resonant PES expressed as a coverage of the surface by Ce^3+^ ions. Lines represent guides to the eyes from **a** and indicate the Pt^2+^ concentration. The Ce^3+^ concentration is lower or equal to the Pt^2+^ concentration on all samples.

The amount of Pt^2+^ ions as a function of the step density is plotted in Fig. 2a. For the higher Pt coverage 0.18 ML, the analysis reveals a linear dependence between the amount of stabilized Pt^2+^ ions and the step density (Fig. 2a, blue symbols), confirming the activation of Pt oxidation to Pt^2+^ and the localization of Pt^2+^ at the surface steps. The highest step density 0.20 ML allows converting up to 80% of the Pt deposit to Pt^2+^. The degree of oxidation of the Pt deposit increases with decreasing the amount of deposited Pt to 0.06 ML (Fig. 2a, black symbols). In this case, up to 90% of Pt converts to Pt^2+^. The concentrations of ceria step edges and the amount of Pt^2+^ stabilized on the surface obey a classical supply-and-demand scenario characteristic for single-atom catalysts^1^,^4^,^10^: when sufficient step edges, the amount of oxidized Pt^2+^ is limited by the amount of deposited Pt. Otherwise, the amount of oxidized Pt^2+^ is limited by the step density regardless of the amount of deposited Pt. The Pt deposit exceeding the available step sites cannot be oxidized and nucleates as metallic Pt^0^ clusters on the surface. Besides the high oxidative power of the step edges towards Pt, our quantitative analysis reveals also the capacity of ceria step edges to accommodate a high density of Pt^2+^ ions. Up to 0.16 ML of Pt^2+^ ions can be stabilized by the sample containing 0.18 ML of Pt deposit and 0.20 ML of steps (Fig. 2a, blue symbols). This corresponds to 80% of the step-edge sites being occupied by Pt^2+^. In the whole range of the step densities between 0.06 and 0.20 ML, the occupation of the step-edge sites by Pt^2+^ varies between 50 and 80%.

### Stability and charge state of Pt^2+^/CeO_2_(111) samples

The necessity of annealing the Pt deposit on ceria in UHV at 700 K for obtaining Pt^2+^ stabilization in our experiment indicates the activated nature of Pt segregation at the ceria steps and oxidation, and implies considerable thermal stability of Pt^2+^ ions on ceria. High-temperature annealing represents a prerequisite for obtaining Pt^2+^ ions also in the experiments on large-area nanostructured ceria samples^3^,^4^. Once created, Pt^2+^ ions remain stable on repeated annealing at 700 K in UHV. The Pt^2+^ ions in our experiment also remain stable on adsorption and thermal desorption of CO in UHV (Supplementary Fig. 2), or on exposure to air at ambient conditions (Supplementary Fig. 3).

Parallel to the charge state of the Pt deposit we determine the charge state of the CeO_2_ support, in particular the concentration of surface Ce^3+^ ions that is indicative of reduction of the ceria surface. Contrary to the case of stabilizing Ni^2+^ ions on ceria^21^, we observe that Pt oxidation during annealing is not accompanied by a corresponding reduction of CeO_2_(111) surface (Fig. 2b). This rules out the direct participation of ceria into the observed Pt oxidation at steps and indicates the involvement of other oxidizing agents in the Pt^2+^ stabilization, such as excess oxygen atoms. In the UHV environment of our experiments, the eligible source of excess oxygen can be water adsorbing in sub-ML amounts from background atmosphere (Supplementary Fig. 4) and undergoing dissociation on reduced ceria and Pt/ceria substrates^22^,^23^. In the large-area Pt^2+^/CeO_2_ catalysts displaying high concentration of Pt^2+^ ions and exceptional redox reactivity, excess O atoms may be incorporated during the synthesis that proceeds in air^3^,^10^.

### Segregation of Pt at CeO_2_(111) step edges

**Figure 3:**
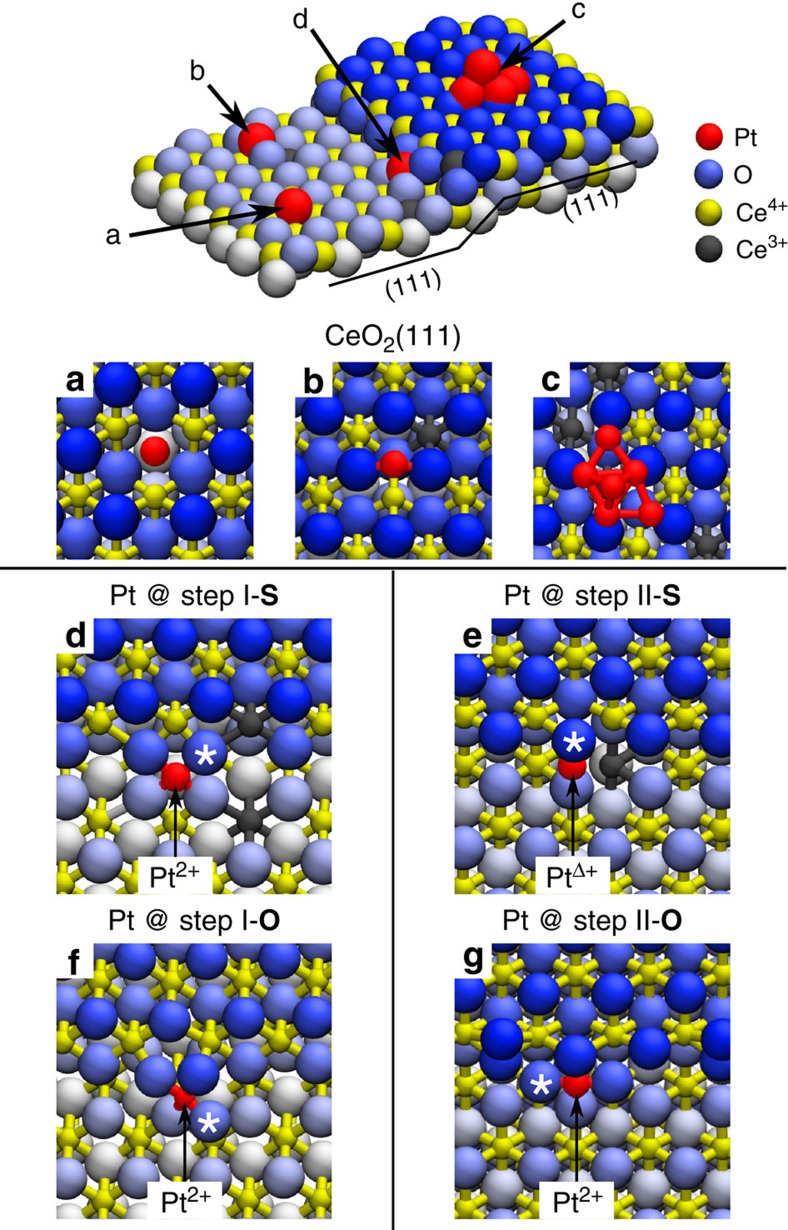
Pt adsorption sites on the CeO_2_(111) surface obtained from DFT calculations. (**a**) Pt adatom in a surface O vacancy, (**b**) on the stoichiometric CeO_2_(111) terrace and (**c**) supported Pt_6_ cluster. (**d**) Pt adatom at the stoichiometric step I (step I-S) and (**e**) at the stoichiometric step II (step II-S). (**f**) Pt adatom at the step I with excess O (step I-O) and (**g**) at the step II with excess O (step II-O). Binding energies and Bader charges are summarized in Table 1. (**d**–**g**) The * symbol denotes the O atom removed to calculate the O vacancy formation energy reported in Table 2.

*Ab initio* density functional theory (DFT) calculations allow to interpret the above experimental results. We calculate the segregation thermodynamics and the atomic and electronic structures of Pt atoms in representative adsorption sites on CeO_2_(111) surfaces. The results for the lowest-energy configurations are summarized in Fig. 3 and Table 1. The model adsorption sites include oxygen vacancies (Fig. 3a), regular sites (Fig. 3b) and Pt clusters^24^ (Fig. 3c) on the CeO_2_(111) terrace, as well as two low-energy ML-high steps, which we label following ref. 25 as step I (Fig. 3d) and step II (Fig. 3e). A detailed list of the systems considered in the DFT analysis is reported in the Supplementary Note 1. The steps I and II represent the preferred types of steps at the CeO_2_(111) surfaces at temperatures <1,000 K (ref. 25). On our experimental samples, the steps I and II appear in equal proportion as evidenced from the absence of triangularly shaped islands in Fig. 1a,g^25^,^26^.

In agreement with the experiment, our calculations predict the preferential segregation of Pt adatoms at the steps I and II, independently on the local step geometry and stoichiometry. The binding energies of Pt at the steps are 1.6–3.4 eV higher than at stoichiometric or defective (111) terraces (Table 1). This driving force for Pt segregation at the steps is in qualitative agreement with recent calorimetric studies of other metal clusters on CeO_2_(111)^27^,^28^, see the Supplementary Discussion. The particular binding energy and the charge state of Pt atoms at the steps depend on both the local step geometry and stoichiometry (Table 1). In the following, we show that a good agreement with all the experimental observations can only be achieved when considering segregation of Pt at steps in the presence of excess O (calculations denoted as O), while Pt segregation at stoichiometric steps (denoted S) exhibits significant discrepancies.

### Pt at stoichiometric CeO_2_(111) step edges

**Figure 4:**
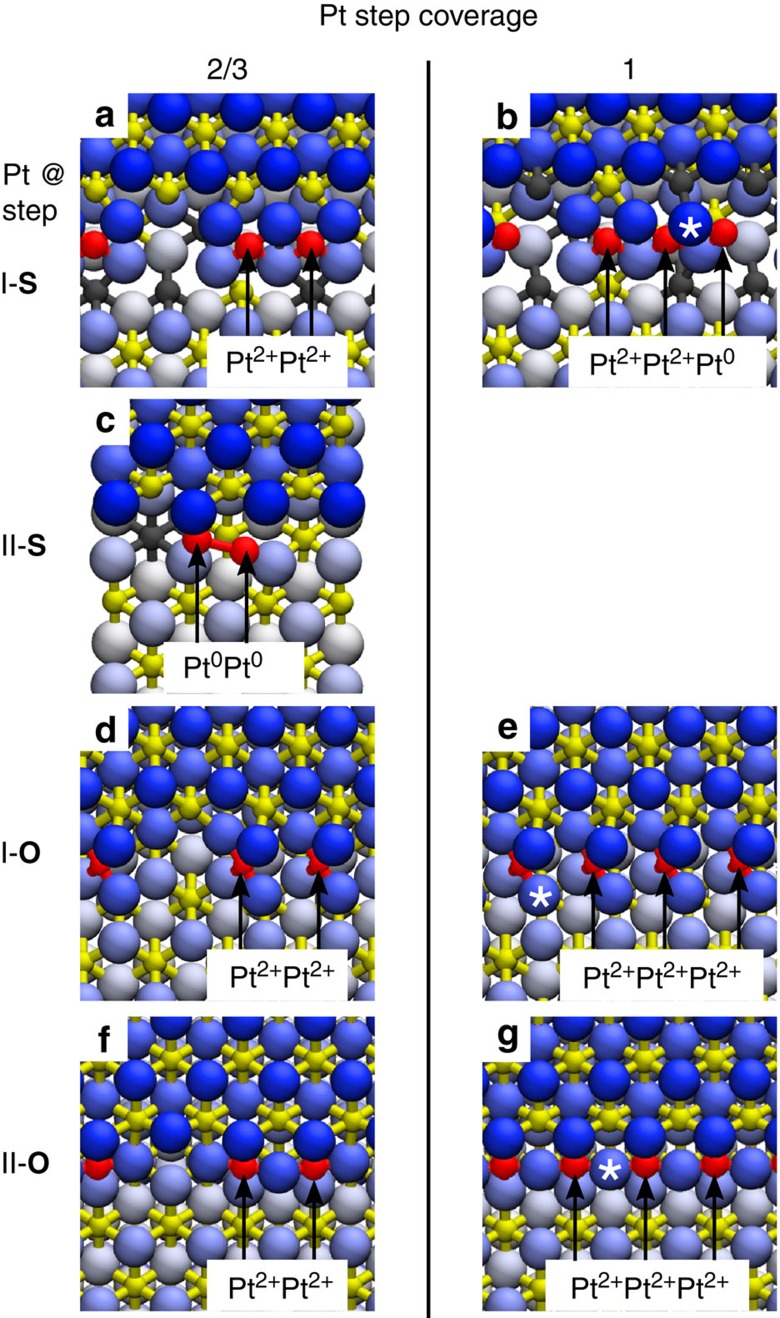
Capacity of the CeO_2_(111) step edges to accommodate Pt^2+^ ions obtained from DFT calculations. Calculated top views of the Pt binding to the steps I-S (**a**,**b**), step II-S (**c**), step I-O (**d**,**e**) and the step II-O (**f**,**g**) for Pt step coverage 2/3 (**a**,**c**,**d**,**f**) and 1 (**b**,**e**,**g**). At the step I-S, the limiting coverage of Pt^2+^ is 2/3 (**a**), additional Pt attaches to step edge as Pt^0^ (**b**). At the step II-S, the Pt^2+^ coverage is 0. Pt atoms attach as weakly ionized Pt^Δ+^ and readily form metallic dimers (**c**) and clusters. On both steps I-O and II-O, excess oxygen can stabilize ionic Pt^2+^ at step edges as single ions appearing isolated or in groups up to 100% step coverage (**d**–**g**). The * symbol denotes the O atom removed to calculate the O vacancy formation energy reported in Table 2.

Pt segregation on step I-S yields Pt^2+^ species that are coordinated by four lattice O atoms in a characteristic PtO_4_ planar unit (Fig. 3d). The PtO_4_ unit is remarkably similar to that one proposed for Pt-doped ceria nanoparticles^4^ and for surface reconstructions of Pd–ceria systems^29^. Instead, the different atomic structure of the step II-S edge prevents the formation of PtO_4_ units, hinders the full Pt oxidation to Pt^2+^ and yields weakly oxidized Pt^Δ+^ species (Fig. 3e). Calculation results presented in Fig. 3d–g correspond to the Pt coverage at the steps 1/3 (1 Pt atom per 3 Ce step-edge atoms). For interpreting the capacity of the ceria step edges to accommodate a high density of Pt^2+^ ions, we calculate the adsorption of Pt at the ceria steps with increasing Pt coverage at the steps (Fig. 4), ranging from 1/3 to 1 (1 Pt atom per 1 Ce step-edge atom). On the step I-S, the maximum coverage of Pt^2+^ species is 2/3 (Fig. 4a). Higher Pt^2+^ coverages are unattainable and lead to nucleation of metallic Pt clusters, due to the large strain buildup resulting from long sequences of interconnected PtO_4_ step units (Fig. 4b). On the step II-S, metallic Pt^0^ species appear already for a coverage higher than 1/3 (Fig. 4c). Thus, on samples with equal proportion of the stoichiometric steps I and II, *ab initio* calculations predict maximum Pt^2+^ coverage at the steps (≤33% of the step-edge sites) and maximum conversion of the Pt deposit to Pt^2+^ (≤33% of deposited Pt) that are well below the experimental values (50–80% of step-edge sites, up to 90% of deposited Pt, *cf*. Fig. 2a).

Most importantly, the calculations on the stoichiometric steps predict that Pt segregation, oxidation and the formation of the Pt^2+^ species are always accompanied by the reduction of surface Ce atoms from Ce^4+^ to Ce^3+^ (denoted in gray in Figs 3 and 4). The resulting concentration of the Ce^3+^ ions exceeds that of the Pt^2+^ ions by a factor of 2. This is in stark contrast with the resonant PES measurements on our samples showing that the concentration of Ce^3+^ is considerably lower than the concentration of Pt^2+^ after annealing the samples (Fig. 2b). This indicates that Pt is preferentially oxidized by other mechanisms than the Pt^0^/Ce^4+^ redox couple.

### Pt^2+^ at CeO_2_(111) step edges with excess O

Agreement between the theory and the experiment can be achieved when taking into account the step edges in the presence of an excess of O atoms. Irrespective of the local step geometry and Pt coverage at the steps, we find that excess O atoms readily bind to Pt at the ceria steps and drive a rearrangement of the step morphology forming ionized Pt^2+^ species incorporated in the planar PtO_4_ moieties on both steps I and II (Fig. 3f,g). In the presence of excess of oxygen, Pt atoms bind stronger to the ceria step edges, with calculated binding energies up to 6.7 eV, which are higher than at the stoichiometric steps edges by ∼1.6 eV, and which are also higher than the cohesive energy of bulk metallic Pt (Table 1). This condition, which determines the stability of the Pt^2+^ species at steps with respect to metallic Pt clusters, is fulfilled only in the presence of excess oxygen at the steps. The computed electronic structure and density of states of the PtO_4_ moieties at the steps I-O and II-O (Supplementary Figs 5 and 6) confirm that the Pt^0^→Pt^2+^ oxidation results from the ionic Pt–O bond in the PtO_4_ planar units, and that Ce^3+^ ions do not form in agreement with the experimental evidence (Fig. 2b). The calculated maximum coverage of Pt^2+^ at the steps I-O and II-O is 100% (Fig. 4e,g and Supplementary Table 2), as interconnected assemblies of the PtO_4_ units can optimally fit the periodicities of both steps I and II at calculated Pt coverages at the step edges 1/3, 2/3 and 1 (Figs 3f,g and 4d–g). The presence of excess oxygen at steps therefore explains also the maximal Pt^2+^ ionization experimentally measured on the ceria-supported catalysts.

The stabilization of excess oxygen in the PtO_4_ moieties by the Pt^2+^ ions suggests an oxygen source for redox reactions and hence provides a link between the presence of highly dispersed ionic Pt species on ceria and the increased redox reactivity of Pt^2+^/CeO_2_ single-atom catalysts^3^. The oxygen buffering capacity of ceria-based catalysts is associated with easy oxygen vacancy formation. We calculate the vacancy formation energy on the clean CeO_2_(111) terrace, on the stoichiometric steps and on the Pt-decorated steps (Table 2). Compared with the energy of 2.5 eV calculated on the CeO_2_(111) terrace, the energies required to remove an oxygen atom bound to Pt at the stoichiometric steps are 3.3 eV (from the PtO_4_ unit at the step I-S) and 3.8 eV (from the PtO_4_ unit at the step II-S; see Table 2). Pt segregation at the stoichiometric step edges yields the formation of strong Pt–O bonds and therefore hinders ceria O-buffering. Much lower energies are instead needed to remove the excess O incorporated in the PtO_4_ units at steps I-O and II-O, where the O vacancy formation energy can be as low as 1.7 eV, lower or comparable to the values for the stoichiometric steps without Pt (2.0–1.8 eV; Table 2)^30^. This indicates that the dispersed Pt^2+^ ions can enhance the oxygen storage capacity of ceria-based catalysts by assisting the reversible storage of excess O atoms.

The single-ion nature of Pt^2+^ in the PtO_4_ units is preserved at all coverages, even when densely packed at the ceria step edges as interconnected PtO_4_ units. Indeed, the Pt charge state, its local electronic structure and the O vacancy formation energy of the densely packed PtO_4_ units are comparable to that of the isolated PtO_4_ units at the steps (Supplementary Fig. 7 and Table 2). The PtO_4_ units exhibit a large adaptability in stabilizing at different types of surface step edges, resulting in high effectiveness and capacity of the ceria surface to accommodate the Pt^2+^ ions. Regardless of the particular organization on the surface, the PtO_4_ units are also always accessible to the reactants. Thus, the square-planar PtO_4_ units carrying monodispersed Pt^2+^ ions can be considered elementary building blocks of single-atom Pt-ceria catalysts.

## Discussion

On large-area samples, Pt^2+^ ions on ceria show exceptional reactivity with minimum Pt load in important applications: water–gas shift reaction^3^, hydrogen oxidation on the anode of proton-exchange membrane fuel cell^11^ and in the three-way catalyst converter^10^. In these applications, Pt^2+^ ions on ceria exhibit long-term stability under realistic reaction conditions of elevated temperatures and ambient pressure of reactant gases^3^,^31^,^32^. Pt^2+^ in large-area samples is routinely identified with PES. Complementary measurements with extended X-ray absorption fine structure (EXAFS)^33^ and high-resolution transmission electron microscopy^3^,^4^,^31^,^32^ confirm the absence of three-dimensional Pt or PtO_*x*_ clusters and, in agreement with the advanced PES measurements^34^, identify Pt^2+^ as highly dispersed surface species on ceria^32^.

Our present study identifies the stabilization of monodispersed Pt^2+^ ions with one particular defect site on the ceria surface—the monoatomic step edge—and excludes the stabilization of Pt^2+^ on the oxygen vacancies. Monodispersed Pt on ceria is observed to be effective in incorporating excess oxygen even in the unfavourable conditions of UHV experiment. Excess oxygen and Pt^2+^ arrange in the square-planar PtO_4_ moieties decorating different types of the surface steps at coverages up to one PtO_4_ per one step-edge Ce atom. The excess oxygen can be easily detached, indicating enhancement of the redox properties of ceria loaded with the Pt^2+^ ions. Adjusting the step density and the Pt load on the model CeO_2_(111) surface allows maximizing the coverage of Pt^2+^, while suppressing the nucleation of metallic Pt^0^ clusters. In the present experiment, we achieve surface coverage of Pt^2+^ 0.05 ML. A further increase of the completely monodispersed ionized Pt^2+^ coverage to 0.1 ML can be expected.

Step edges on ceria have been previously identified as preferred nucleation sites for supported metal clusters^20^,^21^,^27^,^28^,^35^,^36^,^37^. Our present study highlights the property of the step edges on ceria to provide specific structural and electronic environments for selective formation of monodispersed, thermally and chemically stable Pt^2+^ ions. The step edges represent intrinsic defects ubiquitously present on nanostructured ceria surfaces^4^,^38^; our results are thus applicable for the interpretation of the properties and the optimization of the Pt^2+^ load on large-area ceria supports^3^,^10^,^11^. More generally, the step edges may represent a common type of adsorption sites providing stabilization for monodispersed metal atoms and ions in any oxide-supported single-atom catalysts^15^,^39^. Our results therefore introduce important concepts of step reactivity^40^ and step engineering^13^,^14^ in understanding the stability, the activity and in designing new single-atom catalysts.

## Methods

### Experiment

The experiments were performed on surface science apparatuses in Surface Science Laboratory in Prague (STM, laboratory X-ray PES (XPS) with *hν*=1,487 eV (Al Kα), low-energy electron diffraction) and at the Materials Science Beamline in Trieste (PES with *hν*=22–1,000 eV (synchrotron), laboratory XPS with *hv*=1,487 eV (Al Kα) and low-energy electron diffraction).

### Preparation of the ceria substrates

The ceria layers and their Pt loading were prepared using the same procedures and parameters in both laboratories, and investigated by surface science methods *in situ* without exposing to air. The procedures and parameters of all samples are summarized in Supplementary Table 1. The ceria layers were prepared by deposition of Ce metal (Ce wire 99.9%, Goodfellow Cambridge Ltd) from Ta or Mo crucible heated by electron bombardment on clean Cu(111) substrate (MaTecK GmbH) in a background pressure of 5 × 10^−5^ Pa of O_2_ (5.0, Linde AG). The growth rate of CeO_2_ was 6 ML per hour. Varying densities of 1 ML high steps on the prepared CeO_2_ layers were obtained by growth of CeO_2_ at constant substrate temperature 423 or 523 K (Method I in Supplementary Table 1) or linearly increasing substrate temperature from room temperature to 723 K (Method II in Supplementary Table 1)^16^. For experiments in Fig. 1, the ordered, fully oxidized layer of CeO_2_ (Fig. 1a,b) and the ordered reduced layer of CeO_1.7_ (Fig. 1d,e) were obtained by approach published in ref. 17 that yields the lowest step density. In this approach, first, fully reduced Ce_2_O_3_ layer is prepared by depositing metallic Ce on a CeO_2_ layer and annealing in vacuum. Subsequently, the Ce_2_O_3_ layer is exposed to a controlled dose of O_2_ at 5 × 10^−5^ Pa and annealed to obtain desired stoichiometry CeO_1.7_ or CeO_2_ (Method III in Supplementary Table 1). The CeO_2_ surface imaged in Fig. 1g,h was obtained by depositing 0.3 ML CeO_2_ on the CeO_2_(111) substrate as in Fig. 1a, forming small ML-high islands. This homoepitaxy of CeO_2_ on CeO_2_ yields clearly arranged samples with high step density (Method IV in Supplementary Table 1).

### Characterization of the ceria substrates

The thickness of the ceria layers was determined from the attenuation of the substrate Cu 2*p*_3/2_ XPS signal measured at *hν*=1,487 eV. For calculations, we used inelastic mean free path of electrons in CeO_2_ 11.2 Å. The thickness of the ceria layers was set between 20 and 40 Å or 7 and 12 ML with 1 ML corresponding to 3.1 Å, the distance between Ce(111) atomic planes of CeO_2_. In this range of thickness, the coverage of the Cu substrate by ceria ranges between 97 and 100 % (ref. 17). For determining the density of 1 ML high steps, we use a semi-automated procedure when the first step outlines are marked in STM images manually. Step outlines are then mapped onto a properly scaled and rotated hexagonal mesh of surface Ce atoms. The atoms that are closest to the outlines are automatically identified as step-edge atoms and their density evaluated in ML. The error in determining the density of 1 ML high steps is estimated to be ±10 % and is marked in Fig. 2a.

### Preparation of the Pt deposit

Pt was deposited on the ceria layers from a Pt wire (99.99%, MaTecK GmbH) heated by electron bombardment. Pt was deposited on the sample surface at 300 K and subsequently stabilized by increasing the sample temperature to 700 K at the rate 2 K s^−1^. Both Pt deposition and annealing proceeded in the UHV background pressure 5 × 10^−8^ Pa or below. The thermal treatment supports the ionization of Pt to Pt^2+^.

### Characterization of the Pt deposit

The amount of Pt was calculated from the deposition time after calibrating the constant evaporation rate of the Pt evaporator. The evaporation rate was determined by a Quartz Crystal Microbalance and/or in a dedicated experiment from the thickness of 4-ML-thick Pt layers on CeO_2_(111)/Cu(111) determined by attenuation of the substrate Cu 2*p*_3/2_ XPS signal measured at *hν*=1,487 eV. This dedicated experiment was used to correlate Pt evaporation rates between the two experimental apparatuses. For calculations, we used inelastic mean free path of electrons in Pt 8 Å. The fraction of Pt^2+^ after thermal treatment was determined by fitting the ionic Pt^2+^ and neutral Pt^0^ component in the PES Pt 4*f* spectrum measured at *hν*=180 eV (*cf*. Fig. 1c,f,i). The error in determining the Pt and Pt^2+^ amounts on the studied samples is ±20 % and is marked in Fig. 2a. This error represents the calibration error of the Pt evaporation rate.

### Resonant PES

Reduction of the ceria surface after deposition of Pt and thermal treatment was determined with resonant PES of Ce 4*f* state. We determine the so-called resonant enhancement ratio (RER) as defined in refs 41, 42 from measurements of intensities of Ce^3+^ and Ce^4+^ components of valence-band resonant PES Ce 4*f* spectra of CeO_2_ measured off-resonance (*hν*=115 eV) and on-resonance (*hν*=121.4 eV for the Ce^3+^ component and *hν*=124.8 eV for the Ce^4+^ component). The value of resonant enhancement ratio represents an upper estimate of the concentration of Ce^3+^ ions on the ceria surface and is plotted in Fig. 2b^17^,^42^.

### STM imaging

STM measurements were performed with commercial Pt–Ir tips (Unisoku). STM imaging of CeO_2_(111) and Pt/CeO_2_(111) films was available only via unoccupied states. We used sample voltages 2.5–3.5 V and tunnelling currents 25–75 pA.

### Theory

All calculations were based on the DFT and were performed using the spin-polarized GGA+*U* approach^43^, employing the Perdew–Burke–Ernzerhof exchange-correlation functional^44^ and ultrasoft pseudopotentials^45^. The spin-polarized Kohn–Sham equations were solved with a plane-wave basis set and the Fourier representation of the charge density was limited by kinetic cutoffs of 40 and 320 Ry, respectively. The Quantum-ESPRESSO computer package was used in all the calculations^46^. In the Hubbard U term, the occupations of the *f*-orbitals were defined in terms of atomic wave function projectors and the value of the parameter *U* was set to 4.5 eV, following our previous studies^47^,^48^.

### Slab models

The ceria (111) surfaces were modelled with periodic (3 × 3) slabs being three CeO_2_ ML thick and separated by more than 10 Å of vacuum in the direction perpendicular to the surface. The Brillouin zone was sampled at Gamma point. In the present work, we considered two low-energy ML-high steps that we label following ref. 25 as step I and step II. The edge of both step I and step II steps are oriented along the 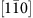 direction. These surface steps were modelled with vicinal surfaces described with monoclinic periodic slabs separated by >10 Å of vacuum in the direction perpendicular to the (111) terrace. The dimensions of the cells were 17.97 × 11.67 Å^2^ along the 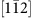 and 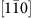 directions (step I) and 15.72 × 11.67 Å^2^ along the 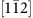 and 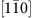 (step II). All the vicinal surfaces slabs included three CeO_2_ ML. This thickness was shown to be sufficient to calculate the structural and thermodynamic properties of these steps^25^. The complete set of surface structures and systems considered in this work is listed in the Supplementary Note 1. All these systems were structurally optimized according to the Hellmann–Feynman forces. During the geometry optimization, the atomic positions of the lowermost CeO_2_ ML were constrained, as well as those of the Ce atoms in the central ML, except for the Ce atoms below the step edge.

### Energetics

Binding energies were computed as 1/*N*_Pt_ (*E*_slab_+*N*_Pt_ *E*_Pt_−*E*_slab/Pt_), where *E*_slab/Pt_ is the total energy of the ceria slab containing *N*_Pt_ atoms of Pt, *E*_slab_ is the total energy of the corresponding relevant (stoichiometric, reduced or oxidized) Pt-free ceria slab and *E*_Pt_ is the total energy of a Pt atom in vacuum. The energies required to form an oxygen vacancy Ov were calculated as (*E*_slab/Ov_+½ *E*_O2_−*E*_slab_), where *E*_slab/Ov_ and *E*_slab_ are the total energies of the ceria supercell with and without the O vacancy, respectively, whereas *E*_O2_ is the total energy of a gas-phase O_2_ molecule compensated for the known overbinding predicted by (semi)local functionals for O_2_.

## Additional information

**How to cite this article**: Dvořák, F. *et al*. Creating single-atom Pt-ceria catalysts by surface step decoration. *Nat. Commun.* 7:10801 doi: 10.1038/ncomms10801 (2016).

## Supplementary Material

Supplementary Information Supplementary Figures 1-7, Supplementary Tables 1-2, Supplementary Note 1, Supplementary Discussion and Supplementary References

## Figures and Tables

**Table 1:** Properties of Pt on CeO_2_(111) obtained from DFT calculations.

**Pt adsorption site**	**Binding energy (eV)**	**Formal charge**	**Bader charge (*****e*****)**
Pt @ O vacancy	2.8	Pt^Δ−^	10.9
Pt @ CeO_2_(111)	3.3	Pt^Δ+^	9.7
Pt_6_ @ CeO_2_(111)	4.4	Pt^0^	9.9
Pt @ step I-S	5.0	Pt^2^^+^	9.2
Pt @ step II-S	5.1	Pt^Δ+^	9.7
Pt @ step I-O	6.6	Pt^2+^	8.6
Pt @ step II-O	6.7	Pt^2+^	9.0
Pt bulk	5.5*	Pt^0^	10.0

DFT, density functional theory; Pt, platinum; I-S and II-S, stoichiometric step types I and II; I-O and II-O, step types I and II with excess O atoms.

Results for the binding energies per atom, formal charges and Bader charges of Pt adatoms at the adsorption sites displayed in Fig. 3a–g. The binding energy for the Pt_6_ cluster is the total binding energy divided by the number of Pt atoms. For reference, Pt bulk values are given in the last line.

^*^Bulk cohesive energy.

**Table 2:** Minimum energy to remove an O atom from the CeO_2_(111) surface obtained from DFT calculations.

**O vacancy site**	**Pt step coverage**	**O vacancy formation energy (eV)**
Pt @ step II-S	1/3	3.8
Pt @ step I-S	1/3	3.3
CeO_2_(111)		2.5
step II-S	0	2.0
Pt @ step I-O	1/3	1.9
Pt @ step II-O	1/3	1.9
step I-S	0	1.8
Pt @ step I-O	1	1.8
Pt @ step II-O	1	1.7

DFT, density functional theory.

Results for steps I and II, and for Pt at steps I and II in the presence and absence of excess O. The energies for Pt @ steps were calculated by removing the O atom marked by the * symbol in Figs 3 and 4.
