# Supplementary material for: Creating single-atom Pt-ceria catalysts by surface step decoration
Source: Nat Commun. 2016 Feb 24;7:10801. doi: 10.1038/ncomms10801 (PMC4770085; doi:10.1038/ncomms10801)
Supplement: Supplementary Information — Supplementary Figures 1-7, Supplementary Tables 1-2, Supplementary Note 1, Supplementary Discussion and Supplementary References [file ncomms10801-s1.pdf]

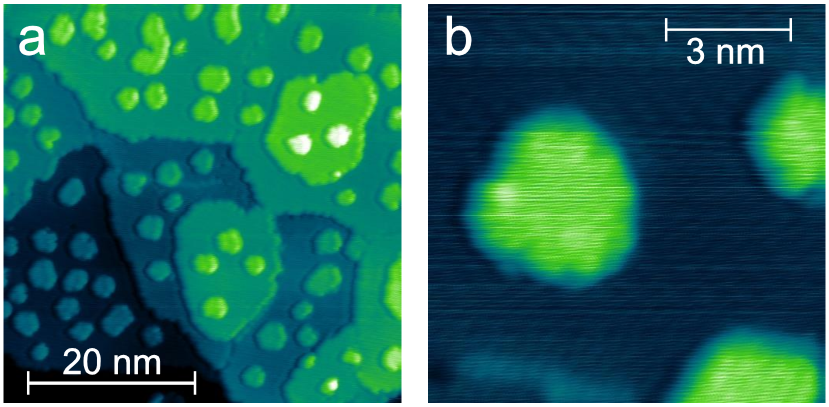

Supplementary Figure 1

Detailed STM images of a CeO_2(111) surface with homoepitaxial islands of CeO_2(111) for increased density of steps. The surface was covered by 0.03 ML of Pt and annealed at 700 K in UHV for stabilizing Pt^{2+}. Expected number of stabilized Pt^{2+} ions is 1000 in (a) and 20 in (b). There are no morphological features detected on different length scales (a), (b), other than CeO_2(111) terraces, step edges and domain boundaries, and no specific contrast changes with respect to the sample without Pt^{2+}. This observation excludes the formation of 3D and 2D aggregates of Pt oxides and proves the incorporation of Pt^{2+} ions in the step edges of CeO_2(111).

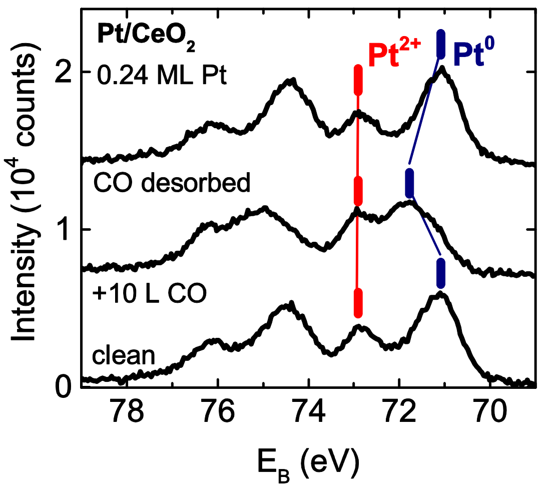

Supplementary Figure 2

Interaction of Pt deposit on the CeO_2(111) surfaces with CO in UHV background. PES Pt 4f spectra of as prepared Pt/CeO_2 (bottom), upon exposure to 10 L CO (middle), and after desorption of CO at 700 K (top), hv=180 eV. Metallic Pt^0 (blue marks) exhibits energy shift due to charge transfer between Pt and adsorbed CO. The state of Pt^{2+} (red marks) remains unchanged indicating no influence of CO on Pt^{2+}.

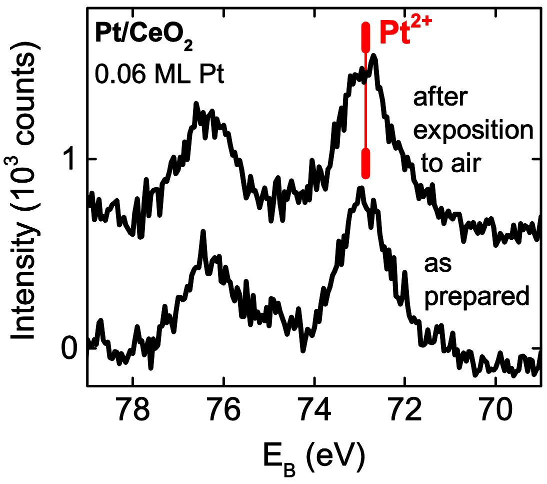

Supplementary Figure 3

Stability of the Pt^{2+} deposit on CeO_2(111) upon exposure to air. XPS Pt 4f spectra of as prepared Pt^{2+}/CeO_2 sample (bottom), and after exposure to air at ambient temperature and pressure, and subsequent evacuation (top), hv=1487 eV. The state of Pt^{2+} (red marks) remains unchanged.

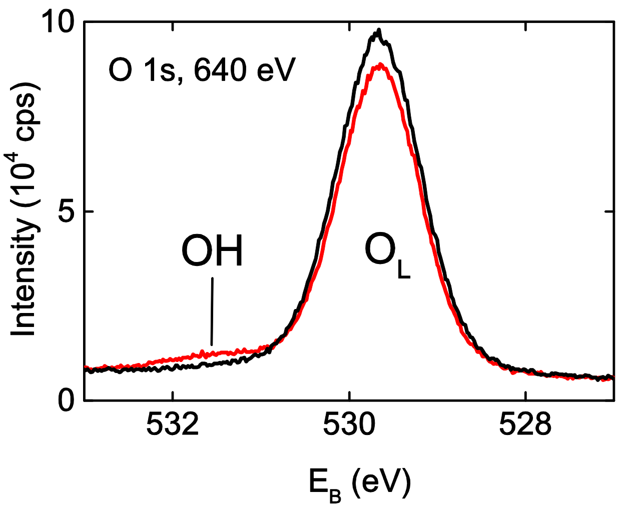

Supplementary Figure 4

Adsorption of water on the samples from residual UHV atmosphere. The adsorption is evidenced from the presence of OH feature in the PES O 1s spectrum after deposition of Pt on the CeO_2 sample (red line). After annealing at 700 K for stabilizing Pt^{2+} adsorbed water reacts off (black line). O_L denotes a peak of lattice oxygen in ceria, hν=640 eV.

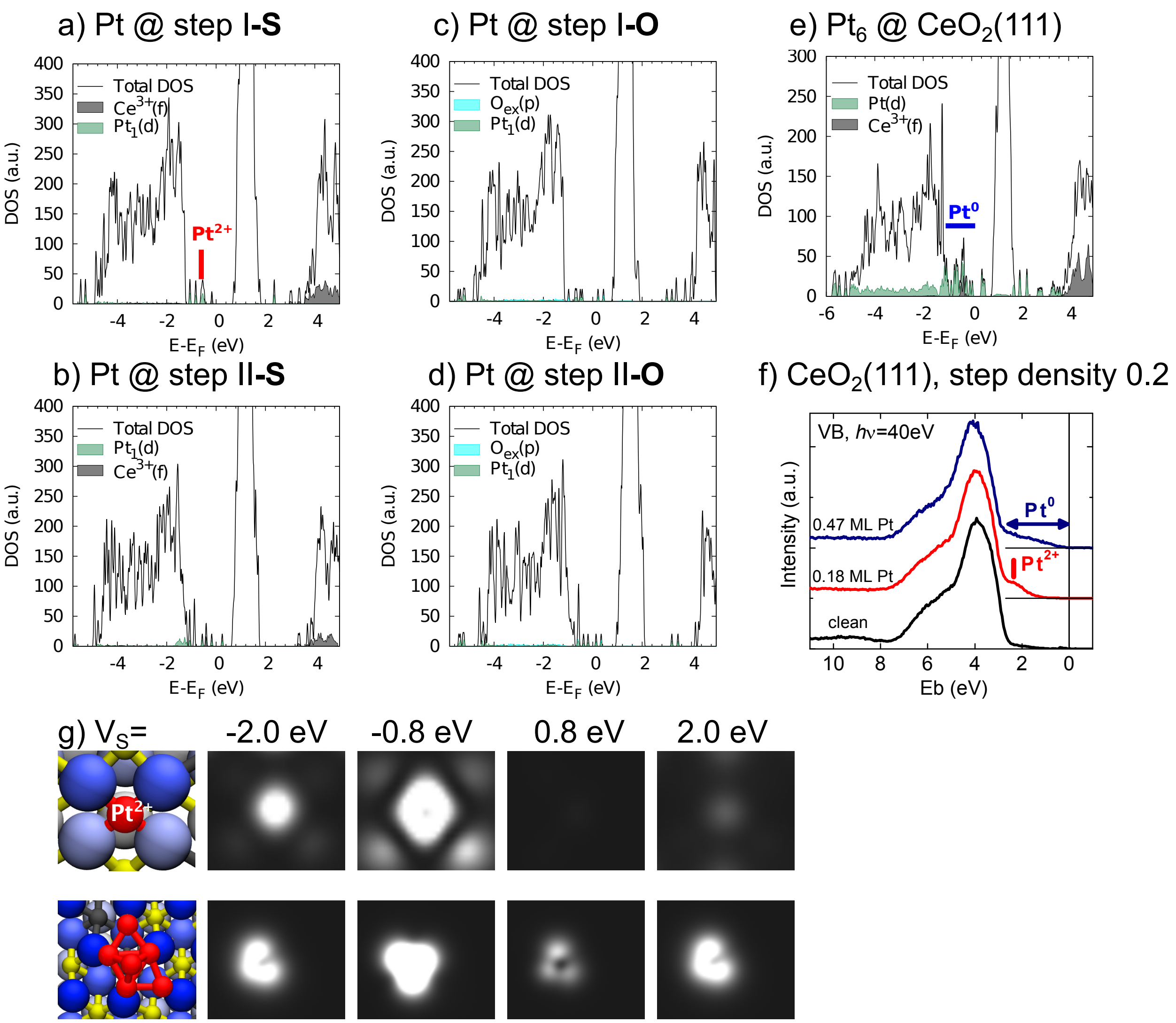

Supplementary Figure 5

Calculated density of states (DOS) and atom-projected DOS (PDOS) for Pt atoms. Results for the steps I-**S**, II-**S**, I-**O**, and II-**O** (a-d, Pt step coverage 1/3), and of Pt_6 cluster on (111) terrace (e). For Pt at the steps I-**O** and II-**O** (c, d) there are no filled Ce f-states, demonstrating the absence of Ce^{3+} ions. Pt atoms at the steps (a-d) exhibit discrete electron states above the valence band (VB) edge of ceria [red mark in (a)], while Pt_6 clusters already exhibit a metallic electron band crossing the E_f [blue mark in (e)]. The calculated VB features of Pt^{2+} ions and of Pt clusters on ceria are observed in PES of VB spectra of Pt/ceria (f) where Pt^{2+} dominates at low Pt coverage (red line), while Pt clusters dominate at high Pt coverage (blue arow). (g) Simulated STM images of Pt^{2+} at step I-**S** and of Pt_6 cluster. Images of occupied states are on the left (V_S= -2 eV, -0.8 eV), empty states on the right (V_S= 0.8 eV, 2 eV). Simulated images of Pt^{2+} in empty states show a negligible STM contrast (cf. Fig. 1 h).

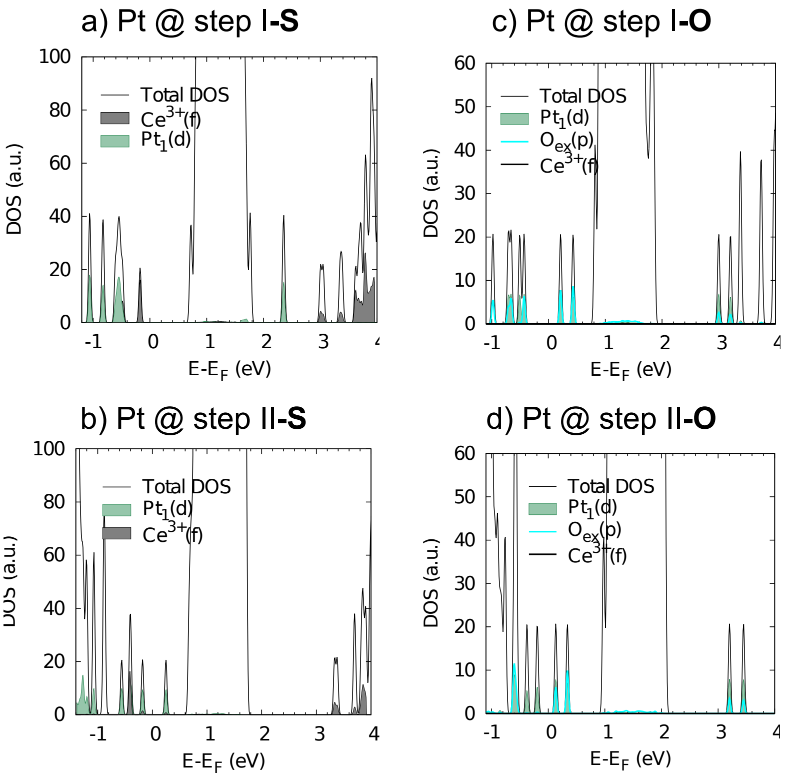

Supplementary Figure 6

Detail of the calculated DOS and PDOS around the Fermi level. Results for the step I-**S** (a), step II-**S** (b), step I-**O** (c), and the step II-**O** (d).

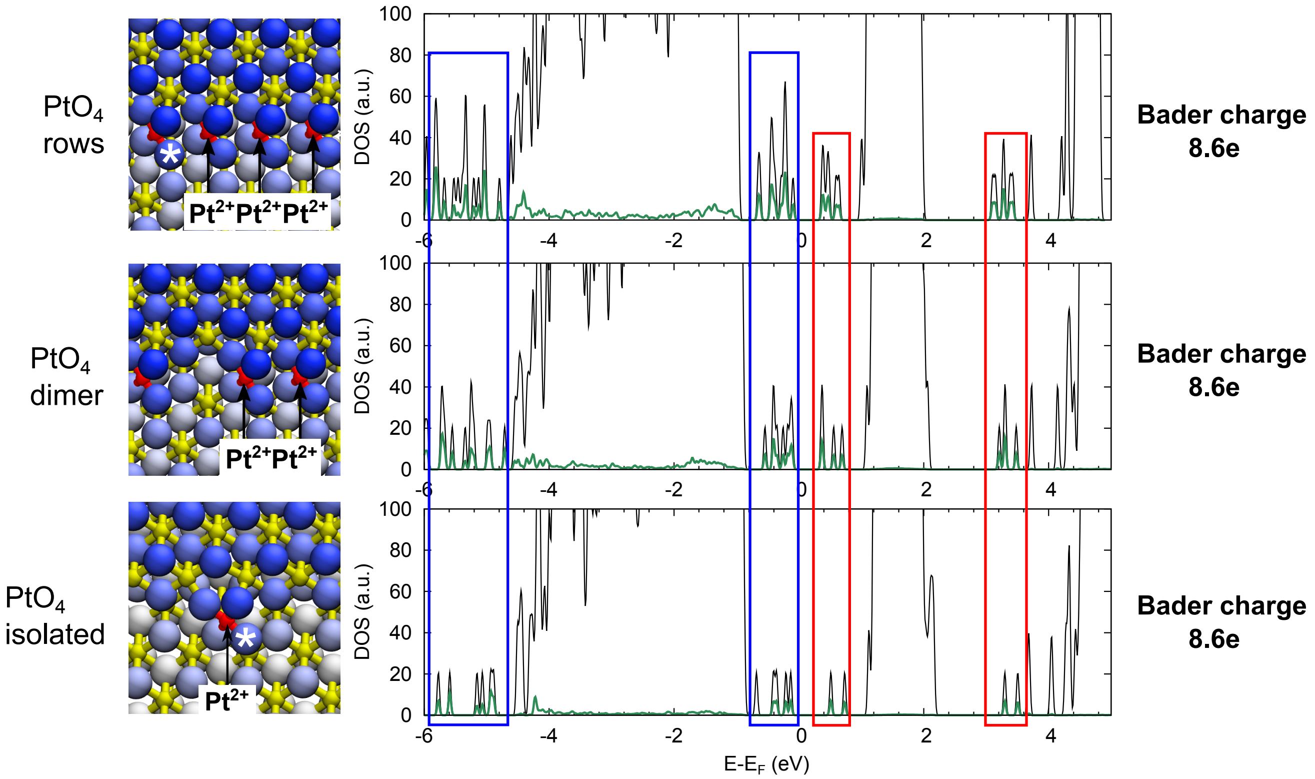

Supplementary Figure 7

Calculated equilibrium geometry, Pt(d) PDOS, and Bader charges for the PtO_4 units. Results for step I-O at coverages 1 (top row), 2/3 (middle row), and 1/3 (bottom row). Black and green lines in the PDOS panels represent the total and the Pt(d) DOS, respectively. The calculated PDOS shows that Pt^{2+} ions in neighboring/interconnected PtO_4 units display the same distinct features in the electronic structure (green lines) both in the occupied (blue rectangles) and unoccupied (red rectangles) states as the Pt^{2+} ions in isolated PtO_4 units.

sample No.	Prep. method	Final prep T [K]	CeO_2 thickness [ML]	[Å]	Step density [ML]	Pt amount [ML]	Pt^{2+} amount [ML]	Ce^{3+} amount [ML]	lab	Figure No. (S denotes Supplementary)
1	I	523	7	22		0.06	0.05	0.03	T	2
2	IV	800	11	34		0.06	0.05	0.02	T	1 j, 2
3	II	723	7	22		0.06	0.05	0.02	T	2
4	III	900	11	34		0.06	0.03	0.03	T	1 c, 2
5	I	423	7	22	0.20	0.06			P	2
6	I	523	7	22	0.16	0.06			P	2, S 3
7	IV	800	12	37	0.15	0.06			P	1 g, 1 h, 2
8	II	723	7	22	0.11	0.06			P	2
9	III	900	11	34	0.06	0.06			P	1 a, 1 b, 2
10	I	423	7	22		0.18	0.16	0.07	T	2, S 4, S 5 f
11	I	523	7	22		0.18	0.13	0.08	T	2
12	IV	800	12	37		0.18	0.10	0.02	T	2
13	II	723	8	25		0.18	0.08	0.04	T	2
14	III	900	11	34		0.18	0.03	0.04	T	2
15	I	423	7	22		0.46	0.22		T	S 5 f
16	II	723	7	22		0.24	0.06		T	S 2
17	IV	800	8	26		0.03	0.03		P	S 1
			**$CeO_{1.7}$ thickness**							
18	III	900	12	37	0.03	0.06			P	1 d, 1 e
19	III	900	10	31		0.06	0		T	1 f

Supplementary Table 1.

Overview of experimental samples. Preparation methods: I - growth of CeO_2 at constant substrate temperature,[1] II - growth of CeO_2 at linearly increasing substrate temperature,[1] III - preparation of fully reduced Ce_2O_3 layer and subsequent oxidation by O_2 to CeO_2,[2] IV - preparation of fully reduced Ce_2O_3 layer, oxidation by O_2 to CeO_2, and subsequent growth of 0.3 ML CeO_2 at 600 K followed by annealing at 800 K in 5×10^{-5} Pa of O_2. Laboratories: T – Trieste, P – Prague.

Pt adsorption site	Binding energy (BE) [eV] Pt^{2+} step coverage 1/3	Average BE [eV] Pt^{2+} step coverage 2/3	Average BE [eV] Pt^{2+} step coverage 1
Pt @ step I-**S**	5.0	4.3	No Pt^{2+}
Pt @ step II-**S**	No Pt^{2+}	No Pt^{2+}	No Pt^{2+}
Pt @ step I-**O**	6.6	6.9	6.9
Pt @ step II-**O**	6.7	6.6	6.2

Supplementary Table 2.

Calculated binding energies (per atom) of Pt^{2+} ions. Results for steps I and II in the presence (**O**) and absence (**S**) of excess O, as a function of Pt step coverage. For step coverages >1/3 we report the average BE of Pt^{2+}.

Supplementary Note 1:

We include in the following list the set of surface sites considered in the computational work.

1. *Isolated Pt adatoms and Pt clusters on flat CeO_2 (111) terraces* (see Figs. 3 a and c). These systems were modeled by adsorbing a Pt adatom and a Pt_6 cluster on the stoichiometric CeO_2(111) surface. The lowest-energy position of the adatom was obtained by sampling the possible symmetry–inequivalent sites while the lowest-energy morphology of the supported Pt_6 cluster was obtained by applying the basin hopping global minimization algorithm. Similar calculations were performed for different cluster sizes, which are not reported in the manuscript text because the main results turned out not to be dependent on the clusters' dimensions. The systems were modeled with a (3×3) periodic supercell. Additional details can be found in Ref. [3];
2. *Isolated Pt adatoms and Pt clusters at O vacancies of the CeO_2 (111) terraces* (see Fig. 3 b). These systems were modeled by creating an O vacancy on the surface of the flat CeO_2(111) surface and by adsorbing a Pt adatom or a Pt_6 cluster at several surface sites, above and nearby the vacancy. In addition, we also considered subsurface O vacancies and other cluster sizes. The systems were modeled with the supercells described above (see point 1). Additional details can be found in Ref. [3];
3. *Isolated Pt adatoms and Pt clusters at the edge of two low-energy surface steps having stoichiometric step edges* (see Figs. 3 d, e; Fig. 4 c;). These systems were modeled as described in detail in the theory methods. In addition to what reported there, we tested the convergence of the calculations against the thickness of the supercell by increasing the number of O–Ce–O trilayers from 3 to 4. For the representative case of Pt @ step I-**S**, these calculations predict a value of the Pt binding energy of 5.2 eV very close to the value of 5.0 eV obtained with the 3-trilayer thick super cell. The size of the Pt clusters at the step edges considered in these cases were limited to Pt_2;
4. *Several coverages of Pt at the stoichiometric step edges* (type I and II) ranging between 1/3 (1 Pt atom per 3 Ce step edge atoms) and 1 (1 Pt atom per 1 Ce step edge atom) (Figs. 3 d, e, Figs. 4 a–c). These systems were modeled with the same supercells described above at point 3. Several possible configurations of the resulting interconnected Pt–O units were explored;
5. *Pt adatoms at the edge of sub-stoichiometric (O vacancies) and over-stoichiometric (excess O atoms) steps I and II* (Fig. 3f,g), *with Pt step coverages ranging from 1/3 to 1* (Fig. 4f-g). These systems were modeled by removing the O atom at the step edges marked with the * symbol in the figures. Several configurations of the resulting interconnected Pt–O units were explored also in this case.

Supplementary Discussion:

The calculated binding energies for Pt on ceria(111) terraces and step edges can be discussed in the broader context of transition-metal adsorption at ceria surfaces. In particular, the binding thermodynamics of the Pt–ceria system displays important similarities and differences with the Cu–ceria systems, which have been recently studied experimentally with calorimetry measurements[4,5]. These works show that O vacancies on the CeO_2(111) terraces are not the trapping centers for Cu adatoms: i.e. Cu binds stronger to stoichiometric surface regions[4]. Our previous DFT calculations are in line with this conclusion, reporting the binding energy (BE) of a Cu adatom to the CeO_2(111) surface lower than to an O vacancy by more than 1.3 eV[6]. A similar trend is obtained in the present work for Pt adsorption on ceria terraces, although the preferential binding to the stoichiometric sites vs. O vacancies (0.5 eV) is reduced with respect to the Cu case (1.3 eV). We find that larger Pt clusters display the same behavior: our DFT calculations for Pt_n (n=2–6) show that metallic clusters bind stronger on stoichiometric surfaces than on O vacancies[3]. A full DFT screening on transition metal atoms at ceria surfaces is under way. We remark that this relative energetics is not general and is reversed for other metals, for example for the case of Au[7,8]. Additional connections can be found concerning the preferential nucleation of metal clusters at the CeO_2 step edges, which is clearly demonstrated by recent calorimetry data and other experiments[4,5,9–13]. For the particular case of the Cu dimer, the experiments determine that the chemical potential of Cu at steps is lower by ~0.6 eV than at the stoichiometric terraces. Here is where some differences arise. In the case of high Pt coverage at the stoichiometric step edges, indeed DFT calculations predict the preferential segregation of metallic Pt clusters at steps. The actual calculated driving force for segregation depends on the type of the step edge and on the size of the cluster. For the specific case of a Pt dimer, the calculated driving force for step segregation is always higher than 1.5 eV/atom, thus higher than the experimentally measured value for the Cu case. As explained in the manuscript, we suggest that Pt step segregation allows for stabilizing a local excess of O atoms at steps, which leads to ionic Pt^{2+} species that are more stable than metallic Pt clusters.

Supplementary References

1. Dvořák, F. *et al.* Adjusting Morphology and Surface Reduction of CeO_2(111) Thin Films on Cu(111). *J. Phys. Chem. C* **115,** 7496–7503 (2011).
2. Duchoň, T. *et al.* Ordered Phases of Reduced Ceria As Epitaxial Films on Cu(111). *J. Phys. Chem. C* **118,** 357–365 (2014).
3. Negreiros, F. R. & Fabris, S. Role of Cluster Morphology in the Dynamics and Reactivity of Subnanometer Pt Clusters Supported on Ceria Surfaces. *J. Phys. Chem. C* **118,** 21014–21020 (2014).
4. James, T. E., Hemmingson, S. L. & Campbell, C. T. Energy of Supported Metal Catalysts: From Single Atoms to Large Metal Nanoparticles. *ACS Catal.* **5,** 5673–5678 (2015).
5. James, T. E., Hemmingson, S. L., Ito, T. & Campbell, C. T. Energetics of Cu Adsorption and Adhesion onto Reduced CeO_2(111) Surfaces by Calorimetry. *J. Phys. Chem. C* **119,** 17209–17217 (2015).
6. Szabová, L., Camellone, M. F., Huang, M., Matolín, V. & Fabris, S. Thermodynamic, electronic and structural properties of Cu/CeO_2 surfaces and interfaces from first-principles DFT+U calculations. *J. Chem. Phys.* **133,** 234705 (2010).
7. Ghosh, P., Farnesi Camellone, M. & Fabris, S. Fluxionality of Au clusters at ceria surfaces during CO oxidation: Relationships among reactivity, size, cohesion, and surface defects from DFT simulations. *J. Phys. Chem. Lett.* **4,** 2256–2263 (2013).
8. Camellone, M. F. & Fabris, S. Reaction mechanisms for the CO oxidation on Au/CeO_2 catalysts: activity of substitutional Au^{3+}/Au^{+} cations and deactivation of supported Au^{+} adatoms. *J. Am. Chem. Soc.* **131,** 10473–83 (2009).
9. Lu, J.-L., Gao, H.-J., Shaikhutdinov, S. & Freund, H.-J. Morphology and defect structure of the CeO_2(111) films grown on Ru(0001) as studied by scanning tunneling microscopy. *Surf. Sci.* **600,** 5004–5010 (2006).
10. Zhou, Y. & Zhou, J. Interactions of Ni nanoparticles with reducible CeO_2(111) thin films. *J. Phys. Chem. C* **116,** 9544–9549 (2012).
11. Zhou, J., Baddorf, A. P., Mullins, D. R. & Overbury, S. H. Growth and Characterization of Rh and Pd Nanoparticles on Oxidized and Reduced CeO_x(111) Thin Films by Scanning Tunneling Microscopy. *J. Phys. Chem. C* **112,** 9336–9345 (2008).
12. Zhou, Y., Perket, J. M. & Zhou, J. Growth of Pt Nanoparticles on Reducible CeO_2(111) Thin Films: Effect of Nanostructures and Redox Properties of Ceria. *J. Phys. Chem. C* **114,** 11853–11860 (2010).
13. Zhou, Y. & Zhou, J. Growth and Sintering of Au−Pt Nanoparticles on Oxidized and Reduced CeO_x(111) Thin Films by Scanning Tunneling Microscopy. *J. Phys. Chem. Lett.* **1,** 609–615 (2010).
